# Integrative analyses of ferroptosis and immune related biomarkers and the osteosarcoma associated mechanisms

**DOI:** 10.1038/s41598-023-33009-1

**Published:** 2023-04-08

**Authors:** Guibin Li, Jie Lei, Dexin Xu, Wenchang Yu, Jinping Bai, Ge Wu

**Affiliations:** 1 Department of Orthopaedics, Jilin Province FAW General Hospital, Changchun, Jilin China; 2 Department of Hospital affairs, Jilin Province FAW General Hospital, Changchun, Jilin China; 3 Department of Drug management, Jilin Province FAW General Hospital, Changchun, Jilin China; 4 Chronic disease outpatient service, Jilin Province FAW General Hospital, Changchun, Jilin China; 5 Department of Clinical Pharmacy, Jilin Province FAW General Hospital, Changchun, Jilin China

**Keywords:** Cancer, Computational biology and bioinformatics

## Abstract

Osteosarcoma (OS) is the most common primary malignant bone tumor with high metastatic potential and relapse risk. To study the regulatory mechanism of the OS microenvironment, a complex regulatory network involving the ferroptosis- and immune response-related genes remains to be established. In the present study, we determined the effect of a comprehensive evaluation system established on the basis of ferroptosis- and immune-related genes on the immune status, related biomarkers, prognosis, and the potential regulatory networks underlying OS based on the TARGET and Gene Expression Omnibus databases that contain information on OS patients by bioinformatics analyses. We first characterized individual ferroptosis scores and immune scores through gene set variation analysis (GSVA) against TARGET-OS datasets. We then identified differentially expressed genes by score groups. Weighted gene co-expression network analysis was performed to identify the most relevant ferroptosis-related and immune-related gene modules, which facilitated the identification of 327 ferroptosis gene and 306 immune gene candidates. A 4-gene (*WAS, CORT, WNT16, and GLB1L2*) signature was constructed and valuation using the least absolute shrinkage and selection operator-Cox regression models to effectively predict OS prognosis. The prediction efficiency was further validated by GSE39055. We stratified patients based on the prognostic scoring systems. Eight hub genes (namely CD3D, CD8A, CD3E, IL2, CD2, MYH6, MYH7, and MYL2) were identified, and TF–miRNA target regulatory networks were constructed. Furthermore, Gene Ontology, Kyoto Encyclopedia of Genes and Genomes, gene set enrichment analysis, and GSVA were used to determine the signature’s potential pathways and biological functions, which showed that the hub genes were enriched in ferroptosis-associated biological functions and immune-associated molecular mechanisms. Thereafter, we investigated the proportion and infiltration extent of 22 infiltrating immune cells by using CIBERSORT, which revealed significant subgroup differences in CD8 + T cells, M0 macrophages, and M2 macrophages. In conclusion, we determined a new ferroptosis-related and immune-related gene signature for predicting OS patients’ prognosis and further explored the ferroptosis and immunity interactions during OS development, which provides insights into the exploration of molecular mechanisms and targeted therapies in patients with OS.

## Introduction

Osteosarcoma, the most common primary malignant bone tumor, mainly occurs in children and adolescents^1^, and its cardinal features include early metastasis, high-grade malignancy, and extremely poor prognosis. Of the patients who develop metastatic disease, 74% experience lung-only metastasis, 9% exhibit bone-only metastasis, and 8% exhibit both bone and lung metastases^2^. Improvement of the 5-year survival rate of OS has been limited over the last four decades^2,3^. Therefore, developing novel therapies to improve patient prognosis is necessary.

Cancer therapy has entered the age of immunity and iron^4^. Cancer treatment has rapidly evolved into immunotherapy, and the effective therapy of established tumors necessitates rational multi-combination immunotherapy including ferroptosis. Recently, many studies have reported the interaction between ferroptosis modifications and antitumor immunity^4–12^. Ferroptosis, characterized by iron-dependent accumulation of lipid peroxides^13^, is a novel cell death pathway and is different from necrosis, autophagy, and apoptosis in cell morphology and function, involving various tumor biological processes such as invasion, metastasis, and immune escape^14^. Ferroptosis and antitumor immunity might synergistically regulate tumor progression in the tumor immune microenvironment (TIME). Ferroptosis of antigen-exposed tumor cells might involve the induction of tumor immunogenicity in TIME^15^. Activated CD8^+^ T cells can facilitate the iron-dependent accumulation of lipid peroxides, further inducing the antitumor effect of immunotherapy^16^. OS is one of the diseases involving the recruitment and activation of tumor-associated macrophages (TAMs) as a treatment strategy. Multiple analyses of the tumor microenvironment of OS indicated that the immune cell infiltrates are composed of macrophages and T cells^17–19^. However, the ferroptosis- and immunity-based predictive biomarkers for the treatment and prognosis of OS patients are not available yet^20^.

Bioinformatics, a novel interdisciplinary subject with rapid development in recent years, reveals the laws and mysteries behind the complex biological data through the comprehensive use of molecular biology, computer science and information technology. Bioinformatics analyses have extended previous works based on screening for predictive biomarkers for ferroptosis and immunity within a variety of disease states.

In the present study, based on bioinformatics, the OS expression data were obtained from the Gene Expression Omnibus (GEO) database and TCGA. The prognostic and diagnostic models were constructed based on the currently available ferroptosis-related genes (FRGs) and immune-related genes (IRGs). Our findings could better elucidate the regulatory network of ferroptosis and antitumor immunity, thereby providing new perspectives on the exploration of molecular mechanisms and novel therapeutic strategies for OS.

## Materials and methods

### Data acquisition and preprocessing

The gene expression profile datasets were downloaded from the TARGET database (https://ocg.cancer.gov/programs/target) from UCSC Xena by using the AnnoProbe package (https://github.com/jiangfuqing/GEO-AnnoProbe) of R software (version 4.0.2, http://r-project.org/). These datasets were set as the training cohort and included 88 samples. The OS expression profile dataset was downloaded from the GEO (https://www.ncbi.nlm.nih.gov/geo/) database and set as the verification cohort, comprising 37 samples of GSE39055 (Table 1). The raw data were read using the affy^21^ package for background correction and quantile normalization. FRGs were acquired from the FerrDb database (https://www.genecards.org/), and IRGs were obtained from the Immport database (https://www.immport.org/).

**Table 1:** Information on the GEO dataset and the TCGA dataset.

Datasets name	GPL number	Chip platform	Group number	Disease type
TARGET-OS	RNA-seq	Illumina HiSeq sequencing	88	OS
GSE39055	Expression microarray	GPL14951: Illumina HumanHT-12 WG-DASL V4.0 R2 expression beadchip	37	OS

### Ferroptosis and immune scores

For all samples within the two datasets, the ferroptosis score and the immune score were calculated by performing a single-sample gene set enrichment analysis (ssGSEA) based on the gene expression matrix by using the R package GSVA^22^.

### Identification of differentially expressed genes

The samples were classified into two groups on the basis of the median values of the ferroptosis score and immune score. Differentially expressed genes (DEGs) in the TARGET-OS dataset were screened using the Deseq2 package^23^, whereas DEGs in GSE39055 were identified using the limma package^24^. The volcano plot and heatmap of DEGs were constructed separately using the ggplot2 package and pheatmap package, respectively. The cut-off thresholds for the two datasets were an adjusted p value of < 0.05 and |log_2_FC|> 1.

### Weighted gene co-expression network analysis

The “WGCNA” package^25^ was used to perform the weighted gene co-expression network analysis (WGCNA) of DEGs. The adjacency matrix was constructed using a weighted correlation coefficient, and the genes had high connectivity in scale-free co-expression networks. Hierarchical clustering trees were constructed based on a high absolute correlation coefficient between the genes. The eigengene was calculated, and the modules were hierarchically clustered. The computational module significance (MS) determines the correlation between ferroptosis and the immune score in different modules. The modules with minimum MS values were considered negative modules, whereas the modules with maximum MS values were considered positive modules. After selecting the modules on the basis of the MS values, all genes within the modules were found to be highly correlated with the ferroptosis and immune scores. Module eigengenes revealed the first principal component in the module and described the expression pattern of the module. The correlation between ferroptosis-related genes, immune score values, and immune genes was determined by gene significance (GS).

### Identification of the prognostic signature genes

In this study, the LASSO regression method was adopted to build the prognostic diagnostic model. Based on the linear regression, we increased the penalty term (the absolute value of the lambda slope), and used the regularization to solve the occurrence of overfitting during the curve fitting process, while improving the generalization ability of the model.A univariate Cox proportional regression model was used to screen candidate genes in the TARGET-OS dataset. The least absolute shrinkage and selection operator (LASSO) Cox regression model was constructed to identify the prognostic signature genes by selecting a standard error above the lowest standard. To make our model more optimized and practical, we used a stepwise Cox proportional hazards regression model.Finally, the risk score was calculated by considering the optimized gene expression and correlation estimated Cox regression coefficients by using the following formula:

Risk score = (exp-Gene1*coef-Gene1) + (exp-Gene2*coef-Gene2) + ⋯ + (exp-Gene*coef-Gene). Patients with OS were divided into high- and low-risk subgroups based on the risk score (median). Kaplan–Meier plots were used to determine overall survival of patients in the two subgroups by using the survival package. By performing a time-dependent receiver operating characteristic (ROC) curve analysis, the predictive ability of the risk score was measured using the ROCR package^26^ to calculate the area (AUC) value under the ROC curve. A prognostic nomogram was constructed to determine survival outcomes based on multiple Cox regression analyses.

Furthermore, the random forest algorithm was used to further validate the accuracy of the model, and we used the Target-OS dataset as the training set and GSE39055 as the test set to further evaluate the reliability of eigengene model building. The AUC values of the ROC curves were performed to assess the accuracy of the model.

### Protein–protein interaction network establishment and hub gene regulatory network analysis

The protein–protein interaction (PPI) network was constructed using the STRING^27^ online tool to examine the interactions between DEGs. The hub genes were further explored using the CytoHubba^28^ plugin in Cytoscape. In addition, the transcription factor-core gene and miRNA-hub gene regulatory networks were constructed using Networkanalyst (http://www.networkanalyst.ca/NetworkAnalyst) and visualized using cytoscape software.

### Functional and pathway enrichment analyses

Enrichment analyses were separately performed for DEGs by referring to the GO and KEGG databases by using the GOplot package^29^. An adjusted p value of < 0.05 was considered statistically significant. GSEA was performed using the clusterProfiler package^30^. “c2.cp.all.v7. 0.symbols.gmt” was selected as the reference gene set, considering that the correlation is significant at a false discovery rate q < 0.25 and a p value < 0.05.

### Correlation analysis of the risk score with immune infiltration

CIBERSORT^31^, a deconvolute method based on support vector regression modeling for 22 immune cell phenotypes in several cancer datasets, determines the abundance and composition of tumor-infiltrating immune cells in complex cells. Gene expression matrix data were uploaded to CIBERSORT, with the absolute level of immune infiltrates within each sample. The immune cell infiltration matrix was derived. Bar graphs were plotted to determine the immune cell infiltrates in each sample by using the R language ggplot2 package. Relevant heatmaps were used to visualize the correlation between the hub genes and 22 immune cell infiltration- or immune-related genes.

### Statistical analysis

All data were analyzed using R software (version 4.0.2). The Student’s *t* test was used to compare the means of the continuous variable with normal distribution. The Mann–Whitney *U* test was used to analyze non-normally distributed continuous variables. The Chi-square or Fisher’s exact test was used to analyze categorical variables. Pearson correlation analysis was performed to calculate the correlation coefficients between various genes. Survival analysis was performed using the R statistical package. Kaplan–Meier analysis was performed to compare survival differences between the subgroups, and the significance was tested using the log-rank test. A nomogram for prognosis prediction was constructed based on univariate and multivariate Cox regression analyses. All P values were two-sided with a significance threshold of 0.05.

### Ethics approval

This study was not conducted on human participants or animals by any of the authors. Our data were downloaded directly from public databases and we strictly abided by the publishing guidelines provided by GEO and TCGA databases; there were no requirements for ethical approvals.

## Results

### Identification of the ferroptosis and immune-related genes

**Figure 1:**
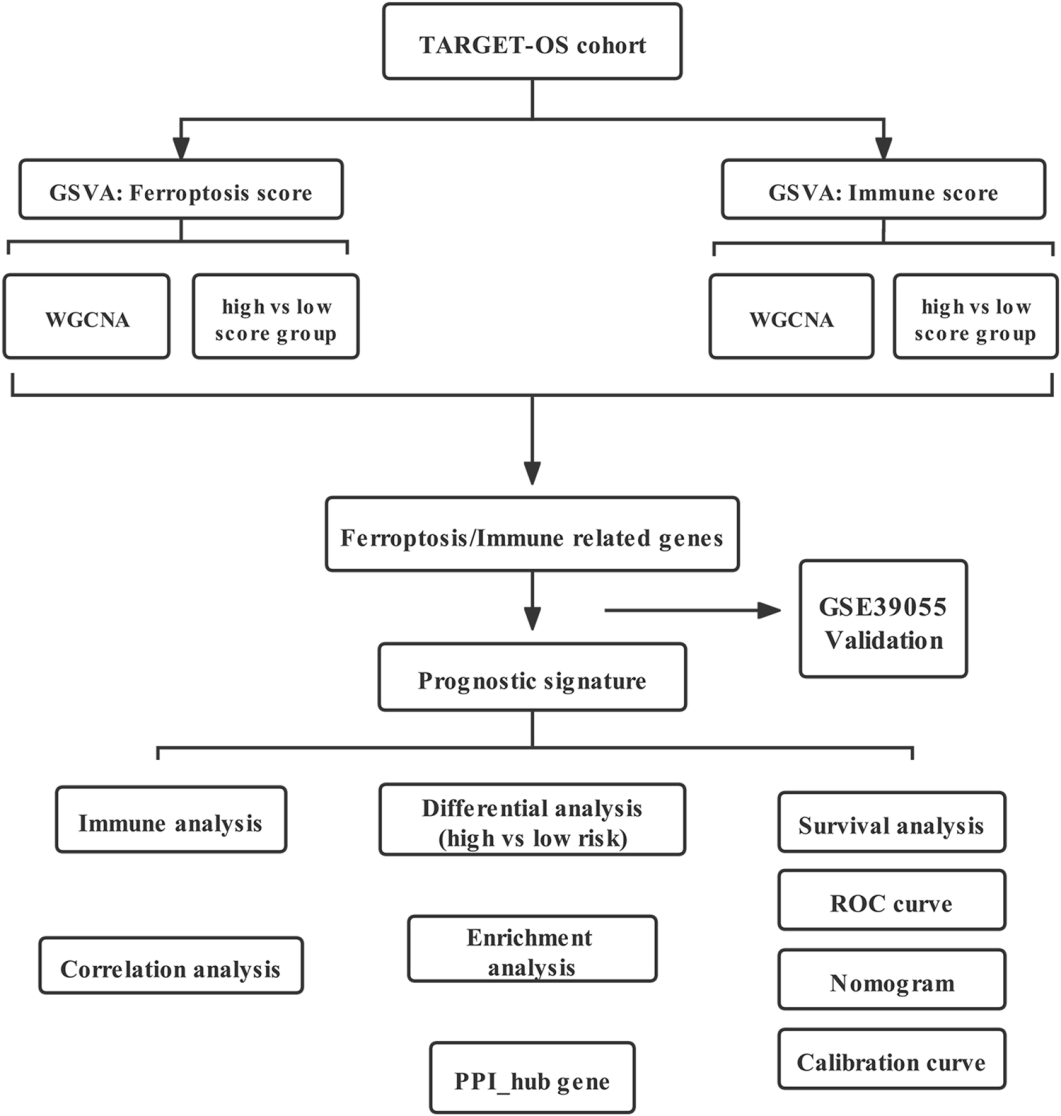
Study flow chart.

**Figure 2:**
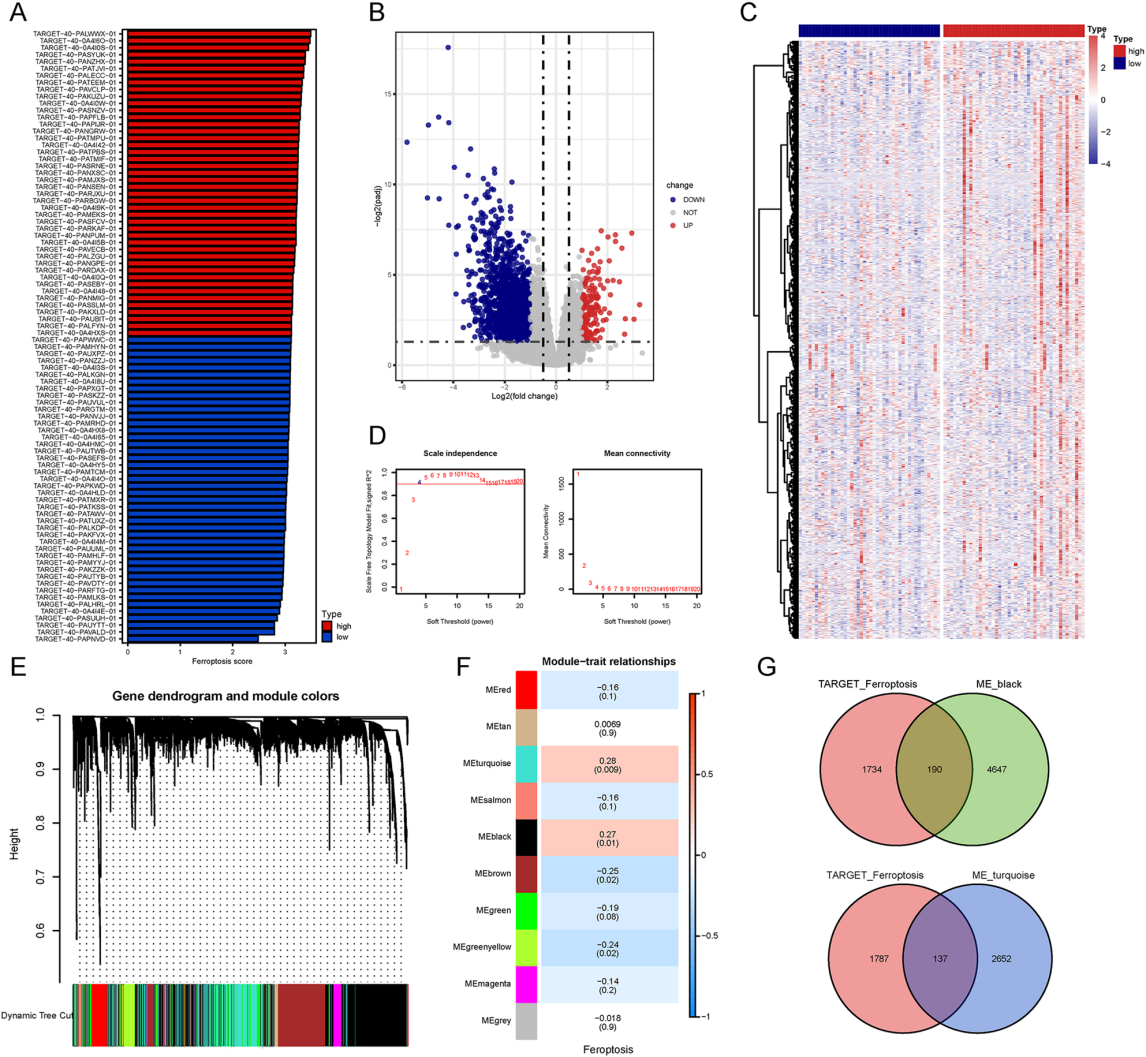
Verification of ferroptosis candidate genes based on the TARGET-OS dataset. (**A**) Overview of the association degree between samples and ferroptosis score from high to low ranking. (**B**,**C**) Volcano plots and heatmaps of differentially expressed genes (DEGs) according to high and low ferroptosis scores. (**D**) Analysis of scale-free fit parameter and mean connectivity for different soft-thresholding power. (**E**,**F**) Dendrograms and heatmaps showing gene modules associated with the ferroptosis score. In Fig. 2F, the p values and correlation values are shown inside and outside the brackets, respectively. (**G**) Venn diagram representing the intersection of genes within the black and turquoise modules, which denote ferroptosis-related genes and DEGs, respectively.

**Figure 3:**
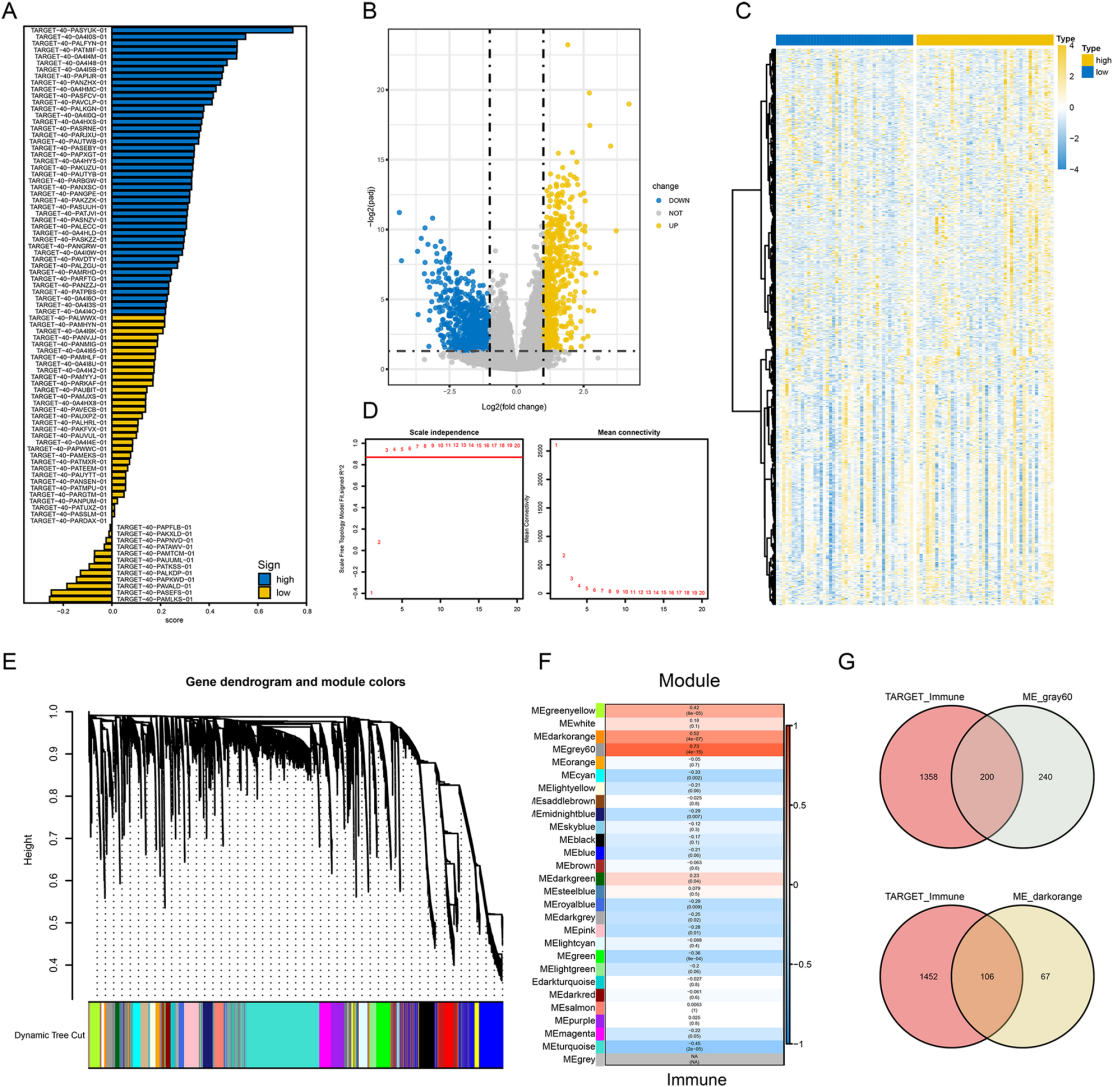
Screening of immune gene candidates in the TARGET-OS dataset. (**A**) Overview of the association of TARGET-OS samples with the immune score. Samples ranked from high to low based on their immune score. (**B**,**C**) Volcano plots and heatmaps of differentially expressed genes (DEGs) according to high and low immune scores. (**D**) Analysis of scale-free fit parameter and mean connectivity for different soft-thresholding power. (**E**,**F**) Dendrograms and heatmaps showing gene modules associated with the immune score. In Fig. 3F, the p values and correlation values are shown inside and outside the brackets, respectively. (**G**) Venn diagram representing the intersection of genes within the gray60 and dark orange modules, which denote immune-related genes and DEGs, respectively.

An overview of the steps is presented as a flow chart (Fig. 1). Transcripts per million-normalized gene expression matrices were obtained using the TARGET-OS dataset. The ferroptosis and immune scores per sample were computed using GSVA. The samples were sorted based on their ferroptosis score and immune score (from low to high) (Figs. 2A, 3A). DEGs were extracted from the gene expression matrix of TARGET-OS by using the DEseq2 package of R software, as indicated in the volcano plots and heat maps (Figs. 2B,C, 3B,C).

Concurrently, we constructed a WGCNA co-expression network to identify biologically significant gene modules and the genes closely associated with ferroptosis and immunity. In this study, 10 ferroptosis modules and 28 immune modules were selected for subsequent analysis (Figs. 2D–F, 3D–F). MS was determined to analyze the association between the ferroptosis and immune scores and genes. As shown in Figs. 2F and 3F, we screened the two most strongly correlated modules in Ferroptosis-related genes and immune-related genes by considering the intersection of all genes of the two modules and the DEGs, respectively, as displayed using Venn diagrams (Figs. 2G, 3G). We obtained 327 ferroptosis-related gene candidates and 306 immune-related gene candidates for subsequent analysis.

### Evaluation of the four-gene prognostic signature

**Figure 4:**
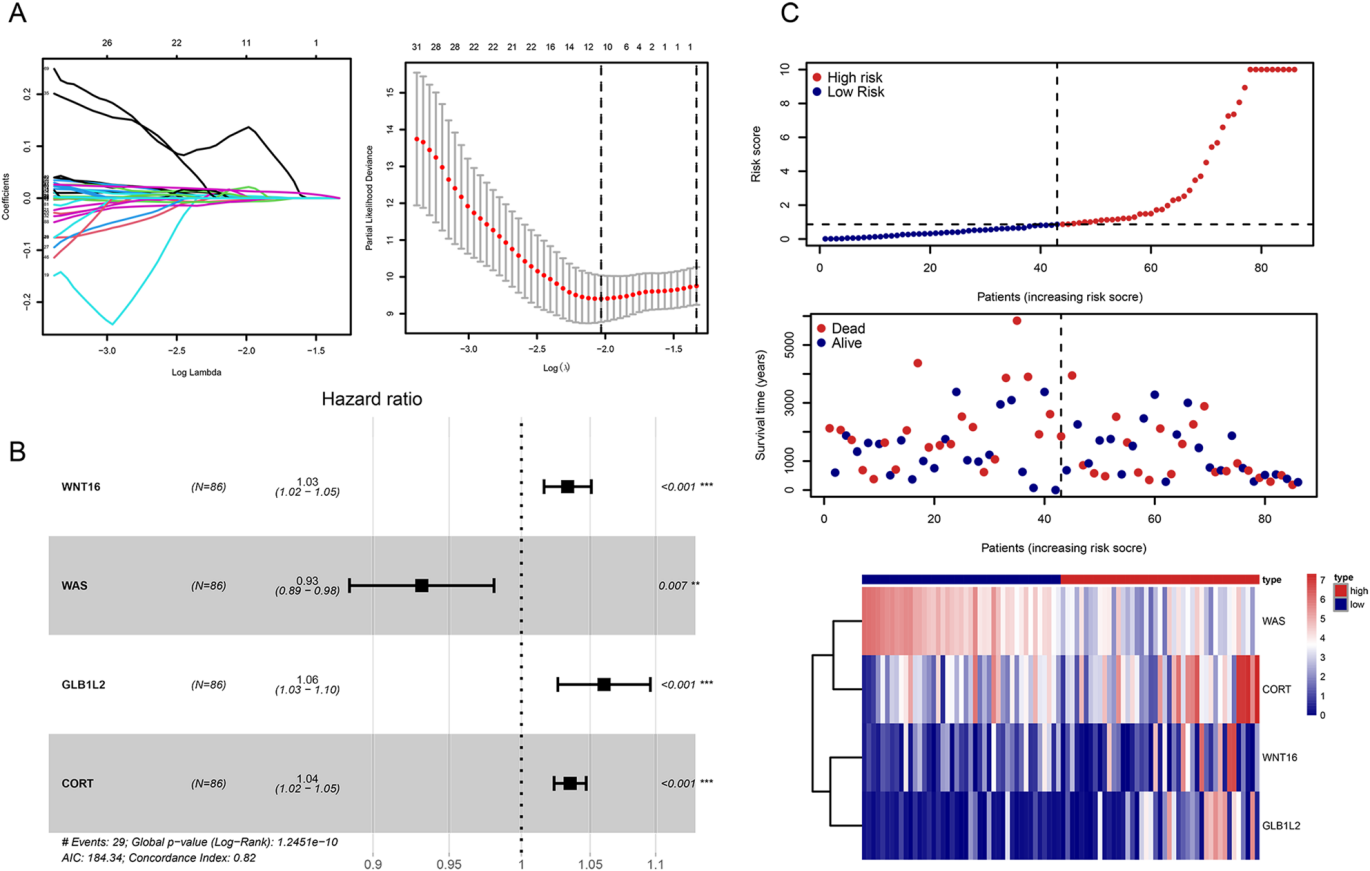
Determination of prognostic ferroptosis- and immune-related genes. (**A**) 100-fold cross-validation for choosing tuning parameters of the Least Absolute Shrinkage and Selection Operator (LASSO) model. The LASSO coefficient profiles of prognostic genes. (**B**) Prognosis-related genes screened further using a stepwise Cox proportional hazards regression model. (**C**) Distribution of the risk score and gene expression profile. The risk factor heatmap shows expression abundance of four hub genes in the high-low riskscore group.

**Figure 5:**
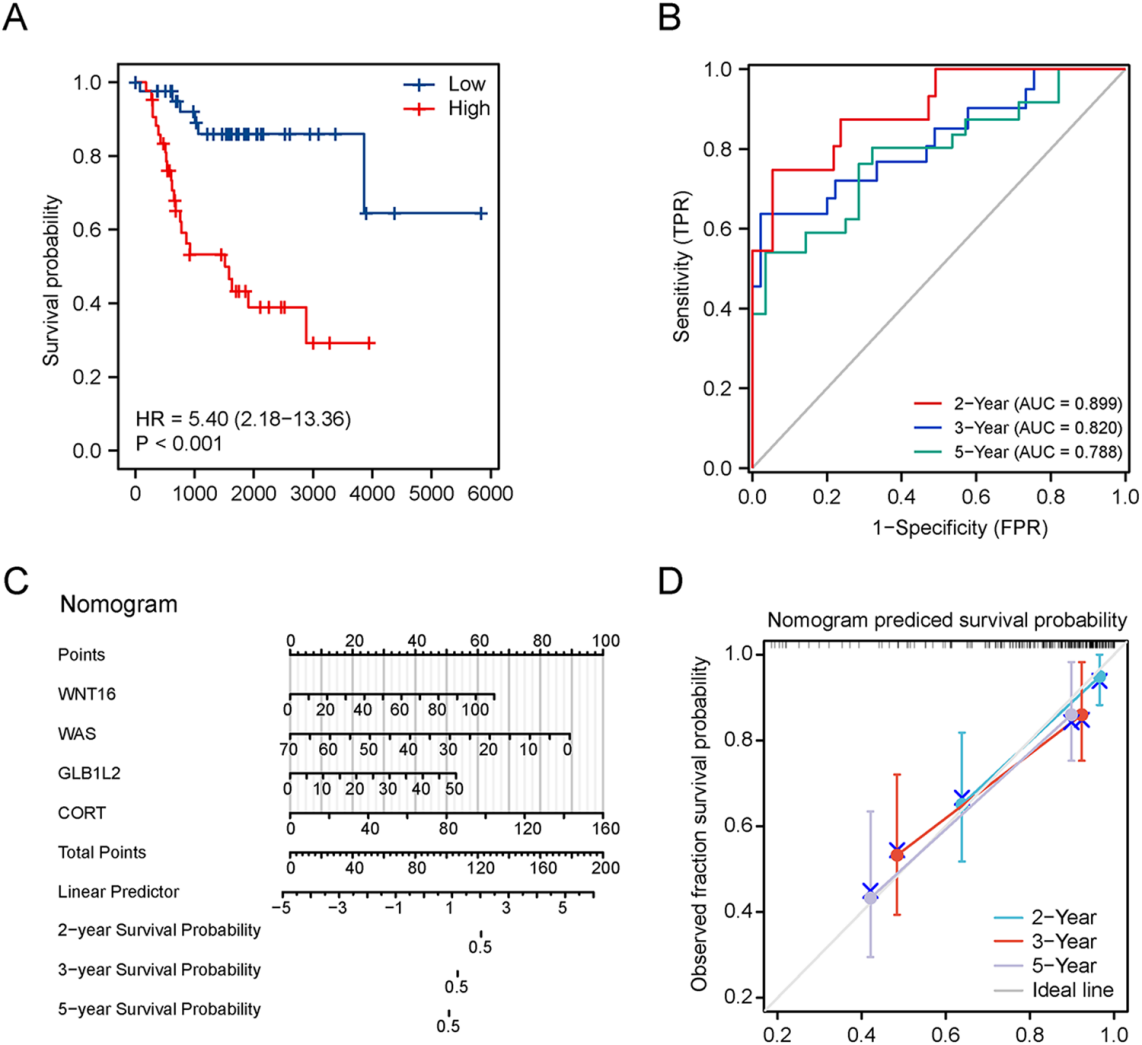
Construction of a prognostic model. (**A**) Kaplan–Meier survival curves indicated that the probability of premature death was higher in high-risk subgroup patients than in low-risk subgroup patients. (**B**) Time-dependent Receiver Operating Characteristic (ROC) curves were plotted to validate the prognostic model, with good discrimination in 2, 3, and 5 years. (**C**) Nomogram based on multivariate Cox regressions to predict the survival probability of OS patients. (**D**) Calibration curves of the nomogram, demonstrating a high prediction accuracy.

After integration, a ferroptosis/immune gene set containing 633 genes was obtained. Among these genes, 131 were associated with OS (P < 0.05). Prognostic risk assessment was performed using LASSO-Cox regression analysis and by establishing a prognostic model based on the obtained gene expression profile (Fig. 4A). 11 genes were obtained after finishing the lasso regression, namely WNT16, WAS, ANXA13, MS4A4A, DLX2, CNNM1, DNAI1, CASP1, GLB1L2, TERT, and CORT. Next, in order to further eliminate the collinearity among genes, we carried out a multifactor Cox regression of 11 genes. The stepwise regression method has three choices: “backward”, “forward” and “both” and we chose “both”. After multiple steps screening in this study, only four signatures were left. To optimize the model, a stepwise Cox proportional hazards regression model was used to identify the final 4 independent prognostic signature genes, namely *WNT16, GLB1L2, CORT,* and *WAS* (Fig. 4B). Subsequently, the patients were categorized into high- and low-risk subgroups (Fig. 4C) based on their optimized predicted risk scores. Patients in the high-risk prognostic subgroup had a significantly higher survival rate than those in the low-risk prognostic subgroup, as represented in the Kaplan–Meier curve (Fig. 5A). The four-gene signature showed significant prognostic effectiveness, with the 2-, 3- and 5-year area under the curve of 0.899, 0.820, and 0.788, respectively (Fig. 5B). Furthermore, through a multivariate Cox regression analysis, a nomogram model was constructed to better estimate the survival rate in OS patients and improve the predictive accuracy (Fig. 5C,D).

**Figure 6:**
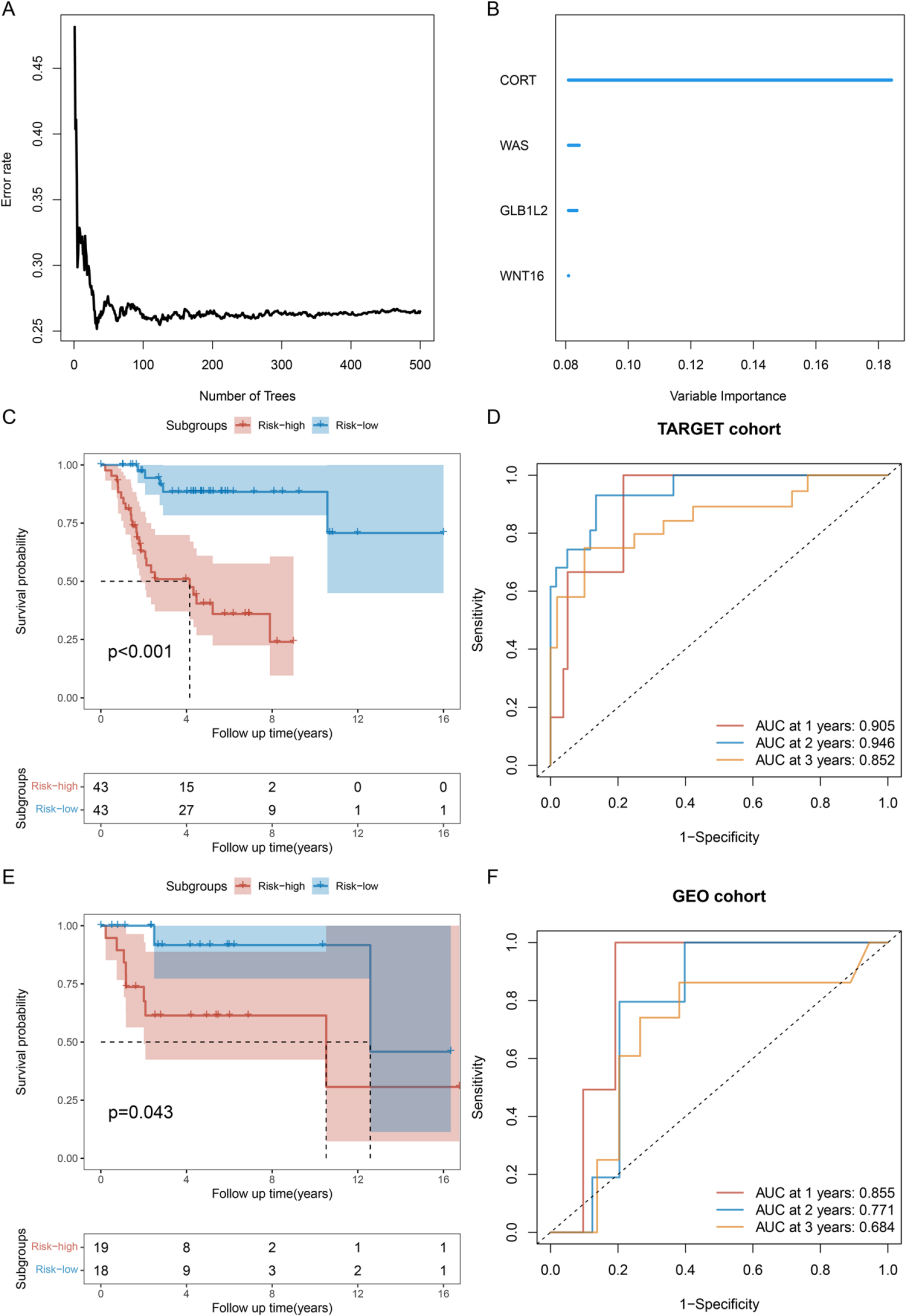
Random forest prediction model construction based on *WAS, CORT, WNT 16 and GLB1L2* eigengenes. (**A**) Training process of the random forest. (**B**) Eigengene importance ranking, importance from large to small order CORT, WAS, GLB1L2 and WNT 16. (**C**) Random forest prediction score based on the TARGER-OS dataset. (**D**) Time-dependent ROC curves were drawn based on the random forest prediction scores of the TARGER-OS dataset. (**E**) Random forest prediction score based on the GSE39055 dataset. (**F**) Time-dependent ROC curves were drawn based on the random forest prediction scores of the TARGER-OS dataset.

We additionally constructed *WAS, CORT, WNT 16 and GLB1L2* as random forest models with eigenes. TARGET-OS was used as the training set and GSE39055 as the test set. The random forest model was trained (Fig. 6A), and it was found that *CORT, WAS, GLB1L2 and WNT 16* were ranked according to the importance of the eigengene (Fig. 6B). Dividing patients into high risk and low risk groups, the training set TARGET-OS results showed significant differences, KM analysis and higher score group found worse prognosis, and time-dependent ROC curve showed the model with 1, 2, 3 year AUC values of 0.905, 0.946 and 0.852, respectively (Fig. 6C,D). Then, the results of the test set GSE39055 met the results of the training set, performed KM analysis and found significantly worse prognosis of patients in the high risk group, and the time-dependent ROC curve showed higher accuracy of the test set model, 1, 2, 3 years AUC values of 0.855, 0.771 and 0.684, respectively (Fig. 6E,F). In conclusion, we verified the possibility of *WAS, CORT, WNT 16 and GLB1L2* as feature genes by Lasso-Cox and random forest model, and both Cox and random forest models showed high accuracy.

### Verification of prognostic genes in the GEO dataset

**Figure 7:**
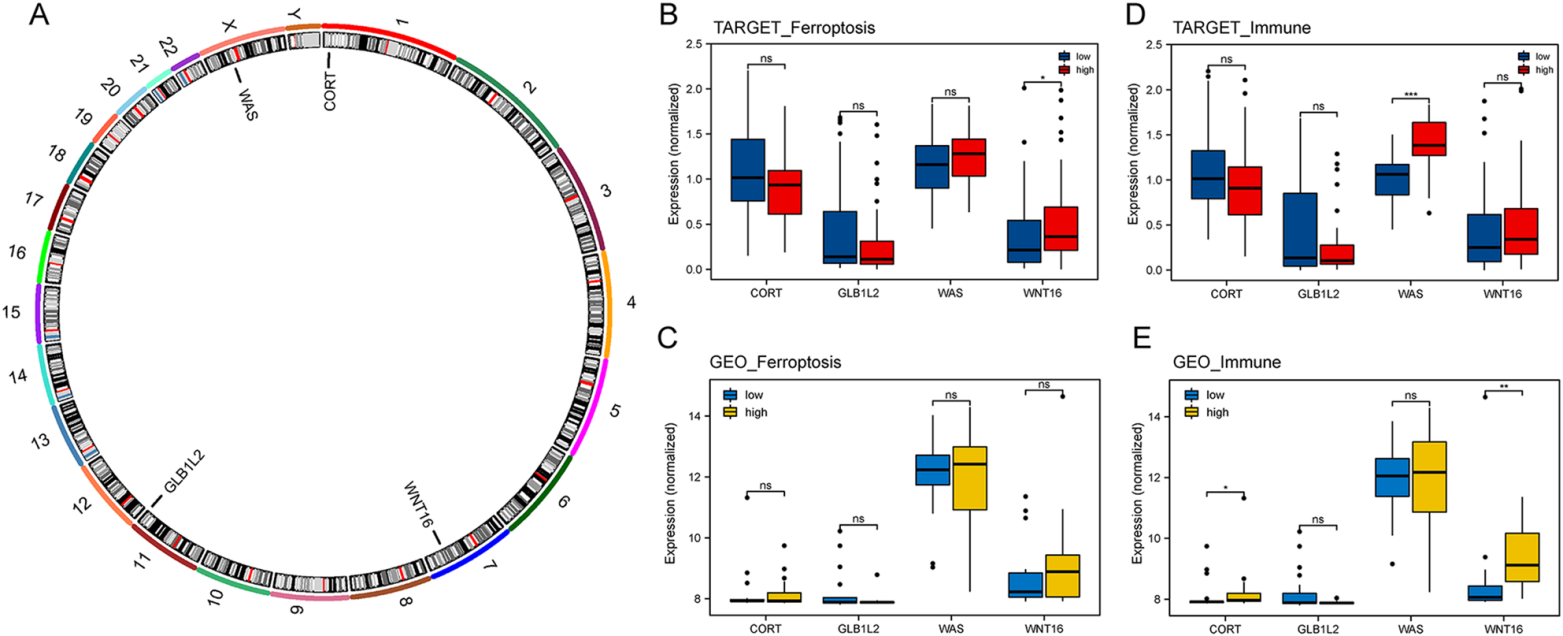
Validation of prognostic gene expression in the TARGET-OS and GSE39055 datasets. (**A**) Positioning of prognostic genes on the chromosome. (**B**,**C**) Differential expression of prognostic genes in high- and low-risk ferroptosis score groups. (**D**,**E**) Differences in the expression of prognostic genes between the high- and low-risk immune score groups.*p < 0.05, **p < 0.01, ***p < 0.001, ****p < *0.0001.*

The expression of 4 independent prognostic signature genes was analyzed using multivariate Cox regression in the TARGET-OS and GSE39055 datasets. The results showed the chromosomal locations of the 4 prognosis signature genes (Fig. 7A). The expression of *WNT16* and *CORT* genes in the TARGET-OS cohort indicated a difference in the ferroptosis score, whereas in GSE39055, none of the genes differed significantly in terms of expression, except for *WNT16* gene. In immune score comparisons, significant differences between *WAS* and *CORT* gene expression were observed in the TARGET dataset. However, in GSE39055, *GLB1L2*, *CORT*, and *WNT16* expressions varied significantly (Fig. 7B–E).

### Analysis of hub genes and enrichment based on the risk score subgroups

**Figure 8:**
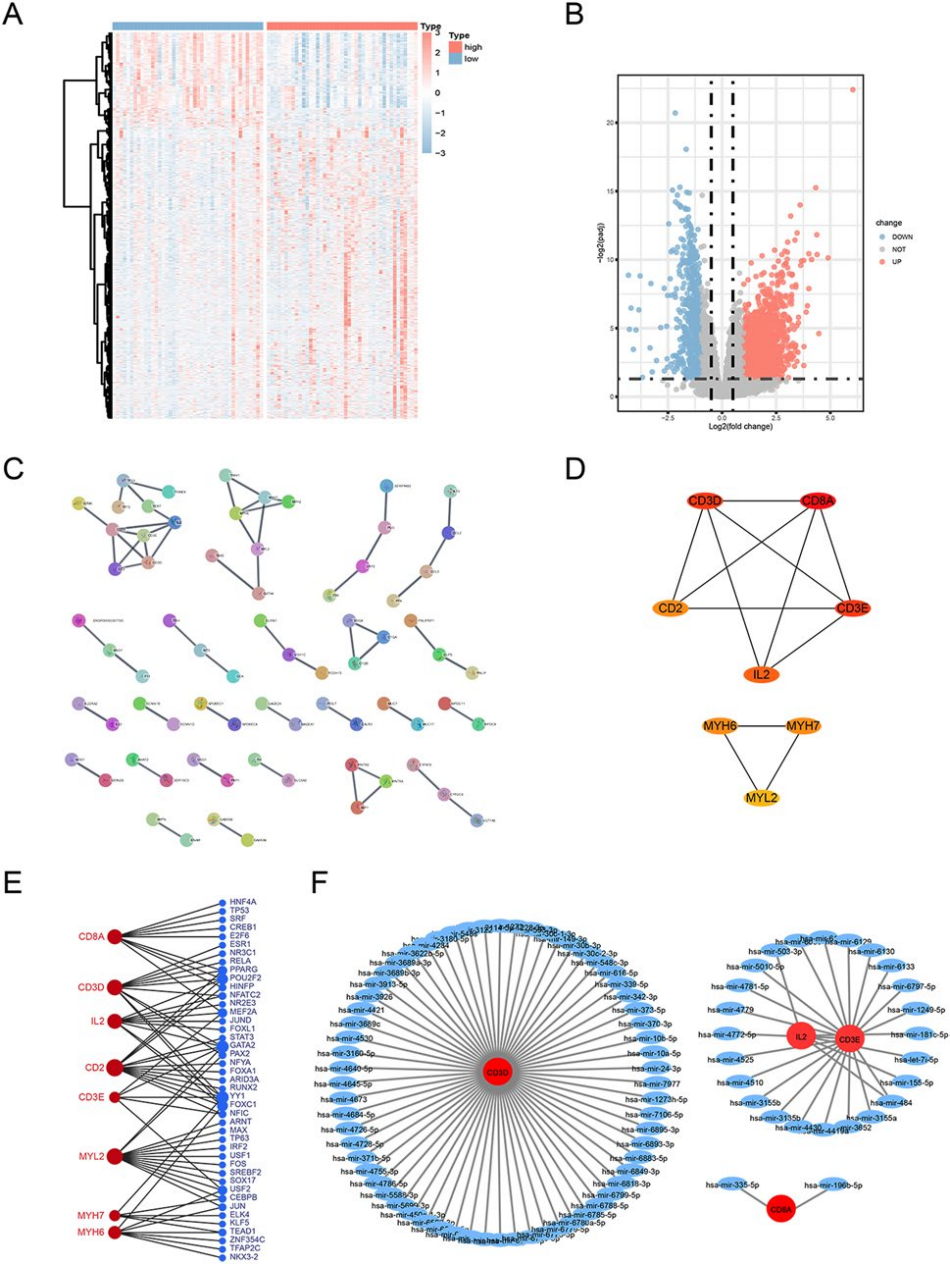
Differentially expressed genes (DEGs) of high- and low-risk groups and regulatory network construction of hub genes. (**A**,**B**) Volcano plots and heatmaps of DEGs according to high- and low-risk scores. (**C**) The DEGs’ protein–protein interaction network was developed using STRING. (**D**) Hub genes were identified using the CytoHubba algorithm. (**E**) Transcription factor regulatory networks of 8 hub genes. (**F**) miRNA regulatory networks of the hub genes.

**Figure 9:**
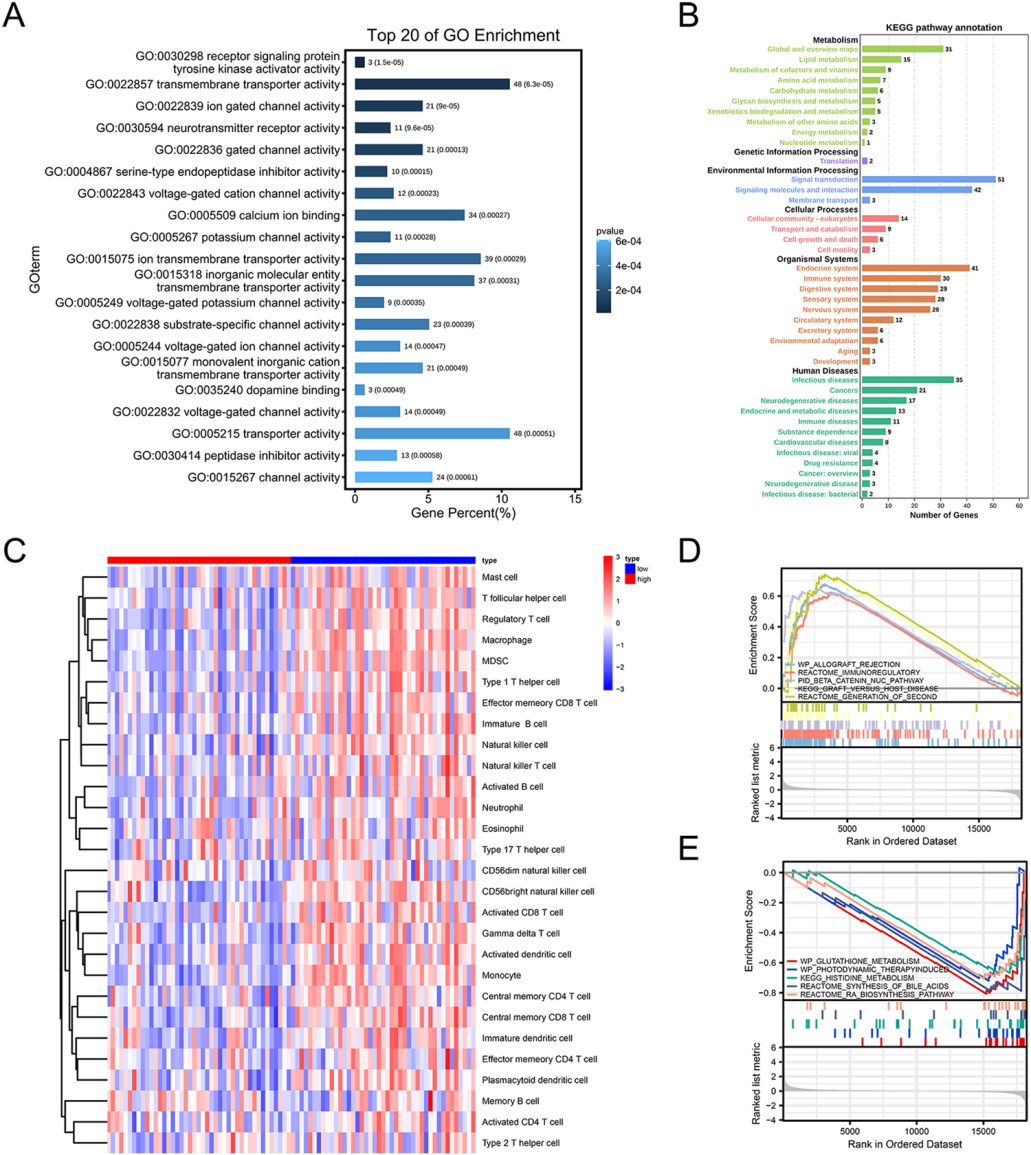
Functional enrichment of differentially expressed genes (DEGs) in the TARGET-OS cohort. (**A**,**B**) Gene Ontology (GO) and Kyoto Encyclopedia of Genes and Genomes (KEGG) enrichment analyses of DEGs in the high- and low-risk groups. (**C**) Enrichment analysis of immune gene sets by using single sample gene set enrichment analysis (ssGSEA) algorithm in the high- and low-risk groups. (**D**,**E**) The enrichment analysis in the high- and low-risk groups through Gene set variation analysis (GSVA) (pathways ranked among top 4 are presented in the results).

Next, we wanted to explore which genes are differentially expressed between the previously identified high and low-risk groups. DEGs from the TARGET-OS gene expression matrix by using R software, as shown in the volcano plots and the heatmaps (Fig. 8A,B). After differential gene expression analysis, a PPI network was constructed using the STRING database. Eight hub genes, namely *CD3D*, *CD8DA*, *CD3E*, *IL2*, *CD2*, *MYH6*, *MYH7*, and *MYL2*, were extracted using the CytoHubba plugin (Fig. 8C,D). As shown in Fig. 7C, when the confidence score > 0.9 (highest confidence), only 72 nodes (genes) existed in the network, and 58 edges (edge) connected them. The average node degree was 0.229, avg. local clustering coefficient 0.113, expected number of edges 18, and PPI enrichment p-value 8.59–14 e-14. Subsequently, we established molecular regulatory networks, which represent the collection of regulatory interactions occurring between transcription factors (TFs)/miRNAs and the identified hub genes (Fig. 8E,F). We further performed GO and KEGG pathway analyses, GSEA, and ssGSEA to identify potential downstream signaling pathways of the 4-gene prognosis model. The GO enrichment analysis showed that the DEGs were significantly enriched in the transporter activity, transmembrane transporter activity, synapse part, sensory perception, etc. (Fig. 9A, Table 2). The KEGG pathway analysis indicated the complement and coagulation cascades, retinol metabolism, neuroactive ligand-receptor interaction, and cGMP-PKG signaling pathway to be the significantly enriched pathways (Fig. 9B, Table 3). Interestingly, ssGSEA indicated that the immune cell infiltration score for the high-risk subgroup was significantly lower than that for the low-risk subgroup (Fig. 9C).GSEA was performed between the high- and low-risk groups in the TARGET-OS dataset to identify the significantly enriched pathways (p < 0.05). We found that WP_ALLOGRAFT_REJECTION, REACTOME_IMMUNOREGULATORY_INTERACTIONS_BETWEEN_A_LYMPHOID_AND_A_NON_LYMPHOID_CELL, PID_BETA_CATENIN_NUC_PATHWAY, and KEGG_GRAFT_VERSUS_HOST_DISEASE were enriched in the high-risk subgroup, whereas WP_GLUTATHIONE_METABOLISM, WP_PHOTODYNAMIC_THERAPYINDUCED_NFE2L2_NRF2_SURVIVAL_SIGNALING, KEGG_HISTIDINE_METABOLISM, and REACTOME_SYNTHESIS_OF_BILE_ACIDS_AND_BILE_SALTS_VIA_24_HYDROXYCHOLESTEROL were mainly enriched in the low-risk subgroup (Fig. 9D,E, Table 4).

**Table 2:** The GO TOP20 analysis of the differentially expressed genes.

ID	p value	Num
GO:0,022,857 transmembrane transporter activity	6.28E−05	48
GO:0,005,215 transporter activity	5.07E−04	48
GO:0,044,456 synapse part	2.05E−04	43
GO:0,015,075 ion transmembrane transporter activity	2.91E−04	39
GO:0,015,318 inorganic molecular entity transmembrane transporter activity	3.08E−04	37
GO:0,004,930 G protein-coupled receptor activity	9.46E−04	36
GO:0,009,986 cell surface	2.76E−03	36
GO:0,005,509 calcium ion binding	2.68E−04	34
GO:0,098,794 postsynapse	3.44E−04	31
GO:0,008,324 cation transmembrane transporter activity	1.45E−03	29
GO:0,015,672 monovalent inorganic cation transport	1.86E−04	28
GO:0,032,102 negative regulation of response to external stimulus	2.12E−04	27
GO:0,098,793 presynapse	5.43E−04	26
GO:0,031,012 extracellular matrix	3.66E−03	25
GO:0,097,060 synaptic membrane	3.66E−05	24
GO:0,015,267 channel activity	6.11E−04	24
GO:0,022,803 passive transmembrane transporter activity	6.11E−04	24
GO:0,022,838 substrate-specific channel activity	3.87E−04	23
GO:1,990,351 transporter complex	4.60E−05	22
GO:0,045,177 apical part of cell	1.23E−03	22

**Table 3:** The KEGG analysis of the significantly differentially expressed genes.

ID	Class	p value
Neuroactive ligand-receptor interaction	Environmental information processing	4.93E−08
Complement and coagulation cascades	Organismal systems	5.16E−05
Fat digestion and absorption	Organismal systems	3.60E−04
Retinol metabolism	Metabolism	1.05E−03
Chagas disease (American trypanosomiasis)	Human diseases	1.05E−03
Salivary secretion	Organismal systems	1.52E−03
Thyroid hormone synthesis	Organismal systems	1.72E−03
Amyotrophic lateral sclerosis (ALS)	Human diseases	2.33E−03
Taste transduction	Organismal systems	2.90E−03
Phototransduction—fly	Organismal systems	3.22E−03
Serotonergic synapse	Organismal systems	6.00E−03
Staphylococcus aureus infection	Human diseases	7.71E−03
cGMP—PKG signaling pathway	Environmental information processing	8.34E−03
Nicotine addiction	Human diseases	1.54E−02
Aldosterone-regulated sodium reabsorption	Organismal systems	1.67E−02
Cardiac muscle contraction	Organismal systems	2.03E−02
Insulin secretion	Organismal systems	2.03E−02
Vitamin digestion and absorption	Organismal systems	2.03E−02
Glutamatergic synapse	Organismal systems	2.22E−02

**Table 4:** Results of the GSEA analysis.

ID	Set size	NES	p value
WP_GLUTATHIONE_METABOLISM	21	− 1.99	2.24E−03
WP_PHOTODYNAMIC_THERAPYINDUCED_NFE2L2_NRF2_SURVIVAL_SIGNALING	23	− 1.82	2.24E−03
KEGG_HISTIDINE_METABOLISM	29	− 1.78	2.26E−03
REACTOME_SYNTHESIS_OF_BILE_ACIDS_AND_BILE_SALTS_VIA_24_HYDROXYCHOLESTEROL	14	− 1.76	2.16E−03
REACTOME_RA_BIOSYNTHESIS_PATHWAY	22	− 1.76	4.49E−03
REACTOME_O_LINKED_GLYCOSYLATION_OF_MUCINS	62	− 1.75	2.22E−03
REACTOME_THYROXINE_BIOSYNTHESIS	10	− 1.75	6.52E−03
REACTOME_REACTIONS_SPECIFIC_TO_THE_COMPLEX_N_GLYCAN_SYNTHESIS_PATHWAY	10	− 1.75	6.52E−03
REACTOME_DIGESTION	22	− 1.75	6.74E−03
WP_NRF2_PATHWAY	143	− 1.74	2.34E−03
PID_GLYPICAN_1PATHWAY	27	1.91	1.75E−03
KEGG_TYPE_I_DIABETES_MELLITUS	41	1.91	1.77E−03
REACTOME_NEGATIVE_REGULATION_OF_FGFR2_SIGNALING	34	1.91	1.80E−03
KEGG_HEMATOPOIETIC_CELL_LINEAGE	84	1.92	1.78E−03
REACTOME_SIGNALING_BY_FGFR4	41	1.92	1.77E−03
REACTOME_GENERATION_OF_SECOND_MESSENGER_MOLECULES	32	1.95	1.80E−03
KEGG_GRAFT_VERSUS_HOST_DISEASE	37	1.96	1.79E−03
PID_BETA_CATENIN_NUC_PATHWAY	76	1.97	1.76E−03
REACTOME_IMMUNOREGULATORY_INTERACTIONS_BETWEEN_A_LYMPHOID_AND_A_NON_LYMPHOID_CELL	127	2.03	1.74E−03
WP_ALLOGRAFT_REJECTION	88	2.10	1.75E−03

### Differences in immune cell infiltration based on risk subgroups

**Figure 10:**
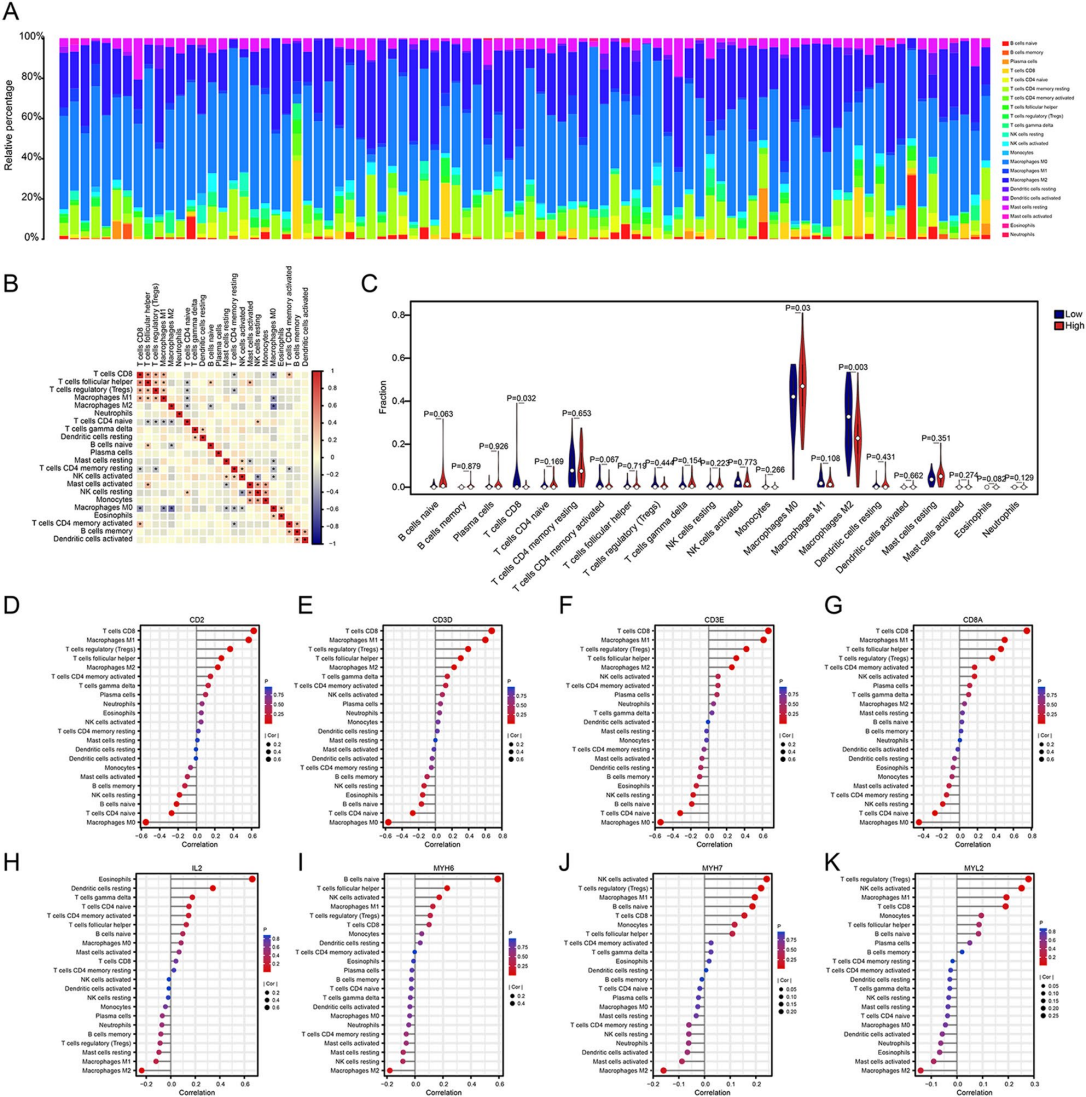
Risk assessment of immune cell infiltration. (**A**) Histograms of the distribution of 22 immune-infiltrating cells in the high- and low-risk subgroups. (**B**) Correlation analysis of 22 immune cells; (**C**) Differential analysis of 22 immune cells in the high- and low-risk subgroups. (**D**–**K**) The correlation between hub genes and 22 immune cells.*p < 0.05, **p < 0.01, ***p < 0.001, ****p < *0.0001*.

Ranked by the risk scores, immune infiltration in TCGA cohorts was shown using bar graphs (Fig. 10A). Correlation analysis (Fig. 10B) of the infiltration score and 22 immune cells was performed on the basis of expression data by using the CIBERSORT algorithm. CD8^+^ T cells, M0 macrophages, and M2 macrophages were significantly different in the high- and low-risk subgroups (Fig. 10C). As expected, the 8 hub genes were closely related to immune cell infiltration (Fig. 10D–K).

## Discussion

Osteosarcoma is the most common bone tumor that affects children and young adults. Despite the emergence of novel therapeutic strategies such as limb salvage surgery, neoadjuvant chemoradiation, and molecular targeted therapies in the last four decades, the survival rate of OS has not improved. The incidences of localized OS or Ewing sarcoma have reached approximately 75%, and that of metastatic disease and initial chemotherapy resistance are less than 30%^32^. Traditional chemotherapy and radiotherapy for OS can result in the development of resistance, which in turn attenuates the therapeutic effects. Recent studies have reported that the long recurrence-free survival rate in patients is approximately 20%^33^. OS development is a highly complicated, multi-step process that involves chromosomal aberrations, genetic mutations, cellular signaling pathways, and epigenetic disorders. However, the molecular mechanism of OS is unclear and the predictive ability of traditional clinical markers is obscure. Bioinformatics approaches provide better insights into the molecular mechanism underlying tumor development and progression by facilitating the identification of new biomarkers that help improve the earlier diagnosis and clinical prognosis prediction of patients with cancer.

Ferroptosis- and immune-related genes and their interactions have garnered considerable scientific attention in the field of cancer-targeted therapies. Ferroptosis, a newly discovered type of cell death, is biochemically and morphologically different from necrosis and apoptosis. Triggering ferroptosis has been confirmed to have therapeutic potential for ferroptosis-sensitive tumor cells. Many studies have extensively explored the involvement of ferroptosis in OS development and treatment^34–37^. Immunotherapy is also an important research area of cancer therapy and has become the most promising therapeutic strategy after conventional cancer therapeutic approaches (such as surgery, radiotherapy, and chemotherapy)^38^. Targeting ferroptosis by inducing peroxidation of lipids in cancer cells provides a novel avenue for anti-cancer therapies. Some studies have reported the adaptation to lethal lipid peroxides of the immune cell in the TME, thus promoting the development of theory and clinical practice for therapies targeting ferroptosis^39^. However, systematic studies combining the two modes for analysis in OS are not available. In this study, we constructed and evaluated genetic diagnosis and prognostic models through the integration and secondary analysis of ferroptosis and immunity, thus revealing the functional role of ferroptosis in tumor-infiltrating immune cells and providing a novel perspective on targeting ferroptosis in OS immunotherapy.

With the influx of tumor tissue sequencing data, the number of available prognostic models is increasing rapidly, which shows the clinical significance of prognosis prediction and potential drugs targeting molecular targets. However, a mature prognostic model for OS is currently unavailable^3^. By using a stepwise Cox proportional hazards regression model, we identified the 4 strongest independent prognostic risk genes, namely WNT16, GLB1L2, CORT, and WAS, and constructed a nomogram model to better predict OS patients' overall survival and verify the prognosis prediction efficiency.

Wnt16 is one of the 19 Wnt ligands in humans, and it has been shown to affect cell proliferation and the TME^40^. As a secreted protein in the TME, it plays a vital role in tumor resistance, metastasis, prognosis, and recurrence by activating the canonical Wnt/β-catenin pathway, which is involved in cell proliferation, migration, invasion, angiogenesis, and immuno-surveillance, thereby enhancing the polarization of M2 macrophages and inducing tumorigenesis^41^. Although the significance of Wnt signaling has been established in epithelial malignancies, its role in mesenchymal tumors is debatable^42^. Some studies have reported that the Wnt/β-catenin-signaling pathway is significantly activated in OS and is associated with tumorigenesis and metastases. Genomic association studies suggest that Wnt16 signaling is a vital mechanism that regulates the bone mineral density, cortical bone density, bone strength, and ultimate fracture event^43^. Even though recent indications that these advantageous effects are coming from WNT-signaling activity, the detailed mechanism of WNT signaling by WNT16 has not been elucidated. One study showed continuous treatment of rWNT16 increases osteogenic differentiation in a c-Jun N-terminal kinase (JNK) pathway-dependent manner. In contrast, WNT16 knockdown significantly reduced PSC osteogenic differentiation^44^, which suggest the MAPK pathway might be an upstream pathway to regulate further the Wnt/β-catenin, the oxidative stress, and the ferroptosis pathways. GPX4 is the ferroptosis core gene, and one recent study speculated that Targeting Wnt/β-catenin signaling by using inhibitors or knocking down TCF4 inhibited the expression of GPX4 and promoted the sensitivity of cancer cells to chemotherapy-induced ferroptosis^45^. Iron dose-dependent downregulation of the expression of WNT target genes disrupts osteoblast differentiation by inhibiting canonical Wnt signaling.Wnt agonist and ferroptosis reverse iron-inhibited traditional Wnt signaling to maintain osteoblast differentiation by reducing reactive oxygen species (ROS) and lipid peroxidation products to prevent ferroptosis effectively without reducing iron overload.The studies above suggest Wnt16 has emerged as a promising target in the field of skeletal research most likely by preventing ferroptosis via canonical Wnt signaling.

Glb1l2 is a member of galactosidase, and its physiological function is largely unknown. A reduction^46^ in *GLB1L2* expression can lead to a decrease in the expression of a tumor-suppressor protein called Inter-Alpha-Trypsin Inhibitor Heavy Chain Family Member 5, which is associated with malignant progression and unfavorable prognosis, indicating a tumor-suppressor function in breast, colon, bladder, hepatic cancers, and lung adenocarcinoma, etc. Jhun et al^47^ reported a reduced *GLB1L2* expression in patients with prostate cancer. The present study observed a correlation of *GLB1L2* expression with OS prognosis and identified *GLB1L2* expression as a risk factor in the prognostic model, which has not been reported in other studies.

CORT, an endogenous cyclic neuropeptide, can modulate inflammatory responses by inhibiting immune infiltration and promoting tumor growth and metastasis. Studies^48^ have reported a high *CORT* expression in high-risk OS, confirming that the high *CORT* expression is associated with adverse clinical prognosis in OS, a finding consistent with that of our study.

WAS gene is localized on chromosome Xp11.23-p11.22, which encodes a 502 amino acid protein (WASp). WASp is a key regulator of the actin cytoskeleton by modulating Arp2/3-mediated actin polymerization, and plays an important role in multiple aspects of immune cell function. Its abnormal function can lead to a variety of immune defects, and patients often show abnormal T lymphocytes, B lymphocytes, and natural killer cell subsets.There are more than 300 known mutations in WAS genes, including nonsense mutations, deletion mutations, insertion mutations, splicing mutations and missense mutations. A WAS gene mutation encoding the WASp causes a rare X-linked recessive disease Wiskott-Aldrich syndrome (WAS), a primary immunodeficiency with microthrombocytopenia and abnormal lymphoid and myeloid functions. As a potential intervention target for immunotherapy, the relationship between WAS genes and osteosarcoma has not yet been revealed.

The prognostic scoring model was developed to classify patients based on their pretreatment risk. Eight hub genes (*CD3D*, *CD8A*, *CD3E*, *IL2*, *CD2*, *MYH6*, *MYH7*, and *MYL2*) were selected for establishing the TF–miRNA molecular regulatory network. The hub genes were highly enriched in immunity, indicating that these genes may be potential therapeutic targets for OS immunotherapy.

CD3D and CD3E proteins, which are present on the T-cell surface, are indispensable for TCR/CD3 complex and contribute to T-cell signal transduction. CD3D is implicated in several cancers, including bladder cancer, colon cancer, breast cancer, and glioblastoma. CD3D is mainly involved in immune activation and regulation and is strongly and positively associated with immune checkpoints. It induces antitumor immunity via the activation of T lymphocytes, particularly CD8^+^ T cells. CD3E, an epsilon subunit of the T-cell antigen receptor, is a new membrane immune biomarker^49^ involved in reshaping suppressive TME by promoting T-cell development and neutrophil activation and mediating the activation of 33 signals of antigens or superantigens. Several studies have reported its overexpression in solid tumors and thus can help improve the prognosis and novel strategies of immunotherapy.

Studies have reported that CD3E affects the TME and the prognosis of patients with cancer, including those with low-grade glioma, cervical squamous carcinoma, head and neck squamous cell carcinoma, and bladder cancer. CD3E tends to show remarkably consistent co-expression patterns across the 31 cancer types. Some novel drugs have been synthesized based on CD3E against breast cancer and solid tumors expressing CEA. Cd2, a cell-surface adhesion protein, is expressed on T cells and natural killer cells. *CD8A* is a key immune activating and cytotoxic gene in OS. The genes *MYH6*, *MYH7*, and *MYH12* might have a relationship with the occurrence and development of heart disease, although their regulatory mechanism in OS is unclear.ssGSEA showed that the immune cell infiltration score of high-risk OS patients was significantly lower. By determining the correlation between the infiltration score of 22 immune cells, we found significant differences in CD8^+^ T cells, M0 macrophages, and M2 macrophages, suggesting that the prognostic model is closely related to the immune status and prognosis of OS patients. The heterogeneous immune microenvironment of OS comprises dendritic cells, natural killer cells, TAMs, and cytotoxic T cells.

OS cells are mainly invaded by TAMs, with M1-polarized macrophages inducing antitumor immunity and M2-polarized macrophages promoting tumor infiltration. M2-polarized macrophages play a key role in tumor progression and metastasis and are associated with poor OS prognosis. TAMs predominantly exhibit the M2-like phenotype in the TME, thereby suppressing the antitumor immunity to promote tumor growth. A study on OS demonstrated that CD163-positive M2 macrophages play a role in inhibiting tumor progression^50^. M2-like TAMs are a contributor to chemoresistance in primary OS cells by inducing IL-1β release. Evidence indicates that all-trans-retinoic acid inhibits tumor metastasis by decreasing the M2-like TAM infiltration and polarization in the TME and pulmonary metastasis in OS patients. By regulating pSTAT3 and IL-10 levels to block TAM-like polarization, dihydroxycoumarins esculetin and daphnetin play an essential role in OS metastasis in vitro and in vivo. In addition, M2 macrophages induce the depletion of T lymphocytes among other tumor-infiltrating lymphocytes, thereby reducing the maturation and secretion of pro-inflammatory cytokines. The results of this study suggested that M2-like TAMs could be a potential therapeutic target for OS.

Many studies have identified a novel mechanism by which CD8^+^ T cells suppress tumors via the induction of ferroptosis and pyroptosis. A study^51^ reported that CD8^+^ T cells trigger ferroptosis by suppressing the expression of *SLC3A2* and *SLC7A11*, suggesting that the immune system might play a role in ferroptosis triggering. OS biopsies have revealed that CD8^+^ cytotoxic T cells are less abundant than myeloid cells, indicating that OS is poorly immunogenic. However, the results of our study indicated that CD8^+^ T lymphocytes are a potential factor for OS prognosis and diagnosis.

This study has some limitations. First, wet lab experiments were not performed; hence, the results of bioinformatics analysis remain to be further validated. Second, the selection of datasets and batch-to-batch variation can inevitably lead to potential biases. Finally, information on comprehensive genomic analyses with clinical correlation could not be obtained. Clinical samples for osteosarcoma are very difficult to obtain, by the bioinformatic approach, screening through global data, is more advantageous for research of rare cases, and in this article, we also verified through the external data set, ensuring the rigor of the data. We will continue to collect blood samples from clinical osteosarcoma over the next few years, expecting to add more clinical validation trials and publish research data. To conclude, the relationship between ferroptosis and OS should be further explored to establish novel treatment strategies for OS patients.

## Conclusion

In this study, we determined the effect of OS prognosis prediction and the TIME from the perspectives of ferroptosis and antitumor immunity by using the comprehensive bioinformatics analysis. Based on the differential modules of FRG and IRGs in OS patients, we established prognostic risk models and multiple target molecules and further found that patients between the prognostic subgroups exhibited different immune states. The findings of this study may contribute to the development of molecular targeted therapy and immunotherapy of OS.

## Data Availability

The datasets generated and/or analysed during the current study are not publicly available but are available from the corresponding author on reasonable request.
